# Stakeholder's perceptions of help-seeking behaviour among people with mental health problems in Uganda

**DOI:** 10.1186/1752-4458-5-5

**Published:** 2011-02-13

**Authors:** James R Nsereko, Dorothy Kizza, Fred Kigozi, Joshua Ssebunnya, Sheila Ndyanabangi, Alan J Flisher, Sara Cooper

**Affiliations:** 1 Department of Mental Health and Community Psychology, Makerere University, Uganda; 2 Butabika National Referral and Teaching Mental Hospital, Kampala, Uganda; 3 Mental Health Department, Ministry of Health Headquarters, Kampala, Uganda; 4 Department of Psychiatry and Mental Health, University of Cape Town, South Africa; 5 Research Centre for Health Promotion, University of Bergen, Norway

## Abstract

**Introduction:**

Mental health facilities in Uganda remain underutilized, despite efforts to decentralize the services. One of the possible explanations for this is the help-seeking behaviours of people with mental health problems. Unfortunately little is known about the factors that influence the help-seeking behaviours. Delays in seeking proper treatment are known to compromise the outcome of the care.

**Aim:**

To examine the help-seeking behaviours of individuals with mental health problems, and the factors that may influence such behaviours in Uganda.

**Method:**

Sixty-two interviews and six focus groups were conducted with stakeholders drawn from national and district levels. Thematic analysis of the data was conducted using a framework analysis approach.

**Results:**

The findings revealed that in some Ugandan communities, help is mostly sought from traditional healers initially, whereas western form of care is usually considered as a last resort. The factors found to influence help-seeking behaviour within the community include: beliefs about the causes of mental illness, the nature of service delivery, accessibility and cost, stigma.

**Conclusion:**

Increasing the uptake of mental health services requires dedicating more human and financial resources to conventional mental health services. Better understanding of socio-cultural factors that may influence accessibility, engagement and collaboration with traditional healers and conventional practitioners is also urgently required.

## Introduction

People suffering from mental health problems very often delay seeking professional help, or avoid it altogether, which in turn significantly compromises appropriate care and treatment [1]. Factors like fear of being diagnosed as suffering from mental illness, distrust towards the system, and lack of confidence in health professionals have been documented to make people hesitant to seek professional help [2]. Seeking help also appears to be related to the individual's perception of the severity of the illness, with individuals who percieve the illness to be severe feeling more compelled to seek help [3]. Furthermore, the choice of where to seek help is said to depend on the what is believed to be the causal factor of the illness [1]. Because mental illness is believed to be due super natural causes, a significant number of people with mental health problems tend to initially seek and to continue seeking traditional healers' services after western medical help [4]. As a result traditional healers find themselves shouldering a large burden of care of patients with mental health problems [5].

Since the initial recognition and response to mental health problems generally takes place in the community [6], Uganda made efforts to bring mental health services closer to the people in the communities [7]. The government of Uganda has taken various steps to improve mental health services in recent years. The Ugandan mental health program was initiated in 1996, and mental health was subsequently included as one of the twelve components of the National Minimum Health Care Package, to be provided at all levels of care [7,8]. Furthermore, working within the Health Sector Strategic Plan, a draft mental health policy was formulated in 2000, which has informed several service reforms within the country [9]. These reforms have made significant steps towards strengthening mental health services in the country. They include decentralization of mental health services; integration of mental health into primary health care, with mental health inpatient units built in each of the 12 regional referral hospitals; and training of primary health care staff in mental health (pre-service and in-service training) [10].

**Figure 1:**
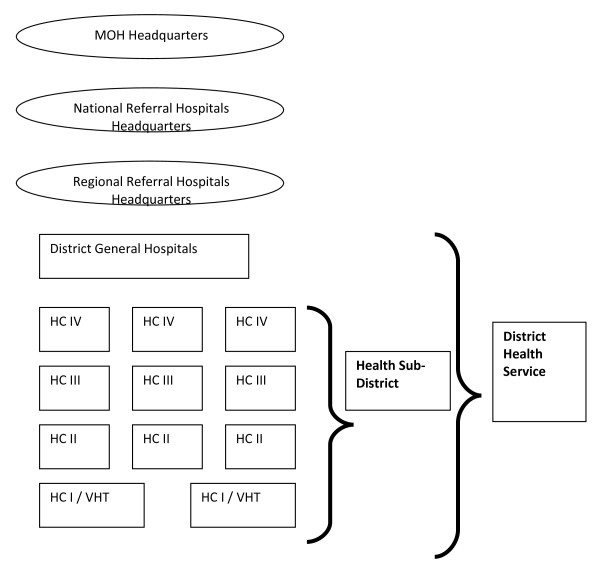
**The National Health system**.

The mental health service delivery system has also been organized in specific levels, so as to ensure that services reach the grassroots. At the apex are the National referral hospitals. Below them are the Regional Referral Hospitals (RRH) which are expected to serve a population of 2,000,000 people. Below RRHs are the District Health Services which are organized hierarchically with a general hospital at the apex, and at the bottom is the health Centre I. Each district should ideally have a general Hospital which acts as a referral point for the facilities with the Health Sub-District. The Health sub-district constitutes of Health Centre IV (County level - 100,000 populations); Health Centre III (Sub-country level - 20,000 populations) Health Centre II (Parish Level - 5,000 population) and Health Centre I (Village Health Team - 1,000 population). The Uganda's health service system is diagrammatically illustrated below in figure 1

It should be noted here that mental health services are provided up to health centre III that is at sub-county level [11]. According to the staffing norms of the ministry of health and the District Service Commission there are stipulated cadres of health workers (mental health workers inclusive) at each level of service delivery. Despite all efforts to bring mental health services closer to the people, such services still seem to be under utilized, particularly in rural parts of the country [12,13].

In this paper, we examine the views of different mental health stakeholders regarding help-seeking behaviours of individuals with mental health problems, and the factors that may influence such behaviours, in the urban and rural communities in Uganda. Such an understanding may help to identify the gaps in service delivery and thus guide the development of relevant services and policies that better meet the mental health needs of the country. This paper forms part of a wider study carried out in several sites (Ghana, south Africa, Uganda, and Zambia), that aims to explore the policy interventions required to address the vicious cycle of mental ill-health and poverty [14].

## Methods

Semi-structured interviews (SSIs) and focus group discussions (FGDs) were conducted with a variety of mental health stakeholders in Uganda. In total, 62 semi-structured interviews and 6 focus group discussions (each consisting of 5-9 participants) were conducted over a 6-month period.

Individual SSIs were used as they are an effective qualitative method for learning about the perspectives of individuals related to a particular topic [15]. These interviews also allowed for the detailed exploration of a particular individual's point of view. FGDs were conducted with some relatively homogenous groups of participants (such as nurses and teachers) in order to elicit participants' views; to document the discussion and interactions between these participants in relation to particular topics; and to capture a range of opinions within these groups, using the limited time and resources that were available.

Selection of the participants was done purposefully, based on two principles: participants represented a range of key mental health organizations in Uganda and they held specialized knowledge on mental health issues. The participants who were interviewed included stakeholders from various sectors as follows:

1. Health Sector (6 Policy makers, 4 Programme managers, 3 Facility managers and 4 health service providers)

2. Education Sector (3 Senior Education Officers and managers, 1 Inspector of schools)

3. Law and justice sector (2 Magistrates and 2 Police chiefs)

4. Gender and Social Welfare department (1 commissioner)

5. Legislators/Politicians (4 Parliamentarians and 1 Minister)

6. Media (3 from print media and 3 from electronic media)

7. Non-Government Organizations in mental health (3)

8. User Support Organizations (1)

9. Academic and Research institutions (4)

10. Housing department (1)

11. Professional Associations (1)

12. External Development Partners/donor agencies (4)

13. Private Sector (1)

14. Religious Leader (1)

15. Traditional Healers (2)

16. Mental Health Service users (7)

The 6 Focus Group Discussions (FGDs) were conducted with homogenous groups, consisting of people of the same background as follows:

1. Mental health nurses, representing 7 health sub-districts in an urban district (7 participants).

2. A mix of general nurses and mental health nurses at the National Mental Hospital (9 participants).

3. Secondary school teachers from 5 schools in an urban district (7 participants).

4. Secondary school teachers from 5 schools in a rural district (8 participants).

5. Primary school teachers from 4 schools in a rural urban district (5 participants).

6. General nurses in a rural district (8 participants)

Of the 106 participants, 48 (45%) were female. The majority of the female participants were from the health and education sectors, specifically nurses and teachers respectively. These two were also the most represented sectors in the study. All the participants were adults aged 19 - 72 years of age; with the majority being in their thirties and forties. All 7 mental health service users were receiving services at the National Mental Hospital, and were mentally well at the time of the study. Four were males.

With the exception of two interviews with service users, the interviews and focus group discussions were conducted in English. The two users who could not speak English fluently were interviewed in the local language, and the interviews were translated to English. Written informed consent was obtained from all the participants. Ethics approval was provided by the Ethics committee and the office of the Director General of Health Services in the Ministry of Health, Uganda as well as the Research Ethics Committee of the Faculty of Health Sciences, University of Cape Town. The interviews and focus group discussions were audio-recorded and transcribed verbatim. The transcriptions were then coded and entered into NVivo7 qualitative data analysis software.

Thematic analysis of the data was conducted using a framework analysis approach [15]. By this approach, certain themes and sub-themes were collectively agreed upon by the investigators at all the research sites, based on the objectives of the study. A single framework for analysis was thus developed, and the transcriptions were coded on the basis of this pre-determined coding frame. Thereafter specific themes emerging from the interviews and focus group discussions were added into the framework in the process of conducting the analysis, and transcripts were coded accordingly. All the four investigators at the Uganda site participated in the coding and analysis of the data. Coding was initially done on paper for the printed transcripts of the interviews. Two of the authors initially did the coding on paper, before data were entered and coded into Nvivo by a third author. The coded material was then checked by partners at Leeds University for consistency.

## Results

In this paper, we present the views and perceptions of all participants as regards help seeking behaviour of persons with mental illness in Uganda. The findings are presented into two parts: i) where help is sought ii) Factors believed to influence help seeking decisions by those people with mental distress and their families.

### I: Where help is sought

Many participants reported that very often help for persons with mental health problems is sought from the traditional healers as a first source, before consulting more conventional Western psychiatric services. One informant estimated up to 65% of people with mental health problems visit traditional healers.

*"...most people here will first try to help themselves.......when they fail, the majority come to traditional healers,.........the biggest percentage of patients (65%) go to traditional healers and they are comfortable with that" (SSI, Key informant traditional healer*)

Similarly, a primary health care nurse explained:

"...*before they come to hospital, they have to try native medicine. They all think they are bewitched. No body ever thinks of going to hospital first..."*

(FGD, PHC nurse 3)

As with these two respondents, many other participants emphasized that "*The first point is always a traditional healer*" and that when people start experiencing mental illness symptoms, they *"always start off by going to a traditional healer".*

This is amplified by a dialogue with this mental health service user, implying that this is the norm:

I: .....now when they started tying you on the ropes...when did your people start seeking treatment for you?

*R: they started right away, but of course they started with the traditional healers *(SSI-Female user).

Some of the patients and their care-takers were said to consider going to a health facility only as a last resort when no improvement is being realized or when the condition is believed to be getting worse.

On those rare occasions where help is first sought from conventional psychiatric services, there was agreement amongst most respondents that the majority of the patients subsequently end up at traditional healers, especially if there is delayed improvement:

"...*the moment a patient stays for a few days without improving, they will immediately say 'ebyekka'*, [Clan Issues] *because I quarreled with so and so. The hospital will not manage'. They run away to a traditional practitioner" *(FGD, PHC Nurse 4, rural district)

The belief in traditional healing was noted to be so strong, that even when traditional healers realize they will not be of help and send patients to health facilities, the patients do not go but instead try other traditional healers only accepting the health facility as a better alternative much later. Seeking help from traditional healers as the first option was reported to apply to other illnesses as well; though very much pronounced with mental illness.

Some of the participants also reported that it is becoming a common practice for many frustrated people to run to churches for consolation and prayers or in the pretext of getting saved, when they are overwhelmed by problems in life. Some users affirmed seeking help from religious leaders and terminating treatment with hope that prayers would bring about permanent recovery.

In addition, it was revealed during the interviews that when families do decide to go to conventional psychiatric services, they usually go straight to higher levels of care such as the regional referral hospital and National Referral Mental Hospital, bypassing primary health care facilities at the local level in anticipation of quality service. This was revealed to occur most commonly in rural areas.

### II: Factors that influence help seeking behavior

Help-seeking was noted to depend on a number of factors such as: beliefs about the causal factors, nature of service delivery, social economic status, severity of the condition, stigma, testimonies from those who have benefited from the services and awareness of the availability of services.

#### Belief system about causal factors

The traditional belief system and cultural explanatory models of mental illness were noted to be very influential in the choice of where to seek help. It was highlighted that mental illness is mostly perceived to be due to witchcraft, curses and evil or ancestral spirits. As a community development officer suggests:

*"...most people think that it is bewitching. Others associate it with disagreements with their elders, for instance we have 'sengas [aunties]' and 'kojjas [uncles]', when they talk ill about somebody and then that person eventually gets a mental problem they say that it is the quarrel he/she had. And also leads not to quickly go for medical attention..." *(Community development officer, rural district)

There was almost unanimous agreement amongst respondents that cultural perceptions' of mental disorders as supernatural, *"spiritual" *illnesses or a product of *"evil forces" *were widespread within Ugandan society. Given such beliefs around the causes of mental illness, many respondents indicated that traditional healers were seen by many individuals in the community to be the most appropriate source of care:

*"....we wanted to take him to hospital, but the parents told us it is culture. That they have demons in the family and don't accept the hospital part of it..." *(Secondary school teacher, rural district).

With this supernatural theory of disease aetiology, many respondents indicated that people lacked faith in the ability of conventional psychiatric treatment to treat and cure mental illness. Traditional healers on the other hand were believed to target the root cause of the illness, and thus possessing much more potential for success, as indicated by this head of medical services, rural district

*"If somebody has say schizophrenia, they think it is a spirit of some kind and... it can only be handled by some spiritual healers, not these 'European medicines' as they put it...'the white man's medicine will just accentuate it...it never heals. It just calms it down. But the other one...it really attacks it, tackles the real cause" *(Head of medical services, rural district)

#### Nature of Service Delivery

Accounts by many respondents indicated that the way in which care is delivered is a major influencing factor in help-seeking behaviour. The widespread choice of traditional healing as a mode of treatment was seen to be influenced by the way in which traditional healers deal with clients. Such practitioners were felt to give a lot of time to their clients. Many respondents spoke about the *"good counselling skills" *of traditional healers, and the *"time and care" *that they give to their clients.

Such characteristics were contrasted with more conventional psychiatric practitioners, who were described as often brief and not inclusive. Many participants reported that conventional medical staff often "*call patients bad names", "spend very little time" *with their patients, do not adequately *"tell them about the condition" *and that they rarely look into the patients' social life or welfare and other problems that might affect their wellbeing. As the Head of medical services, in a rural district remarked:

*"So I think they go there *[to traditional healers] *because they have time for them, for us we don't have time for these people we always think we are busy"*

Furthermore, numerous respondents, particularly mental health service users themselves spoke about the *"hostile" *and unkind manner in which patients are dealt with by conventional mental health practitioners. As one service user lamented:

"...Sorry to comment on psychiatrists but when you are in hospital, instead of calling you by name, they call you 'case', 'this case here', 'this mental case.'... That is not a proper way to address people. Why do you call me case? I have a name. I am not a case and I have a right to be called my name. But because they have an attitude of labeling..... you are being turned into an object by them..."

In contrast, many respondents spoke about traditional healers who listen to their clients' complaints with empathy and unconditional positive regard.

#### Accessibility and cost

It was noted that accessibility of health facilities and financial costs associated with care also influence help-seeking behaviour. High transport costs and other financial implications were reported to frustrate patients and their carers, making them resort to the readily available and affordable traditional healers within their communities:

"*People don't have finances to go to hospitals even when they feel they should go and for us we don't have a district hospital. The so called hospital is a private hospital ...when you go to health centre the drugs are not there. ...if you are an informed person, they will treat you. if you are not they will refer you to Buluuba *[a private hospital in the district] *do you have transport to take you..?... people resort to the local herbs. I think that is why they believe in superstition...*" (FGD - Secondary School Teacher 2, Rural District)

Similarly, a mental health nurse explained:

*"To take a patient to Butabika, you need to hire a special transport, and that person must charge you highly because of the risks involved. So it is not worth it for many people... *(FGD, Mental health nurse 3).

She added:

"*And traditional healers are cheaper than others, it is negotiable...."*

Similarly, when talking about traditional healers, another teacher in a rural district exclaimed:

"There charges are quite user friendly....in most cases it is negotiable...it doesn't necessitate a receipt that if you don't pay you are retained. That is why you find that the biggest percentage of patients rather go to traditional healers and they are comfortable with that"

Furthermore, the widespread availability of traditional healers compared to conventional psychiatric practitioners was noted to fundamentally affect help-seeking behavior. Comments such as this were scattered throughout the interviews:

*"There are so many traditional healers here as compared to the medical workers" *(FGD - Secondary School Teacher 3, Rural District)

Similarly, another respondent added:

*"Every home has a shrine" *(FGD - Secondary School Teacher 2, Rural District)

And a third participant exclaimed:

*"We have very many traditional healers...they are readily available and cheaper" *(FGD - Secondary School Teacher 3, Rural District)

It was further reported that one of the reasons why people frequently go straight to higher levels of care such as the regional referral hospital and National Referral Mental Hospital, was that patients and carers are aware that mental health staff are scarce in primary health care facilities at the local level. There were phrases scattered throughout the interviews that *"there are not enough workers" *the *"workforce is insufficient"*, and that there are *"major human resource problems" *in conventional mental health facilities. This medical doctor summed up the situation when talking about mental health:

''The major challenge is inadequate human resources. You find that most of the health facilities don't have adequate staff. That is a big problem. You find...like Butabika...how many doctors are there to attend to the patients? And it is even worse at other health facilities?"

Some respondents emphasized however that people have misperceptions around the scarcity of conventional health care providers. It was suggested that although there are shortages, there are still more workers than commonly assumed. A few respondents highlighted that the public is not fully aware of the availability of mental health services within other health facilities apart from those at the National Mental Hospital.

#### Severity of the condition

It was also noted that help seeking is influenced by the severity of the disorder. Most participants reported that it's rare for patients to seek help at the health facilities, until they feel the condition is severe. Conditions characterized by frequent relapses were deemed severe and therefore require quick attention whereas seemingly less severe ones were often overlooked. Both caregivers and patients wait until the condition is severe enough, when behavior is deemed out of proportion or when it is frequent. This is somehow related to the perception most people have about mental illness. Unusual behavior is mostly attributed to supernatural spirits. The person is being possessed by spirits. In this case it is hoped to last for a brief period. If the behavior persist or the condition worsens then the explanatory model changes.

#### Testimonies from beneficiaries

Participants reported that care givers become motivated to seek help after seeing and hearing from others who have benefited from treatment. In a discussion with teacher, a teacher who also happens to have a patient with mental illness noted that some caregivers could wait to notice a change in other patients or testimonies from other caregivers for them to make a decision to go to hospital.

*"we started coming to collect medicine. So when we started coming for medicine I started to notice a change in my patient - I also talked to my neighbours, they started to come. They started coming for medicine" *(FGD - Secondary School Teacher 3, Rural District).

#### Stigma

Participants reported high levels of stigma extended to both the patients and their families. There were ubiquitous comments throughout the interviews that people with mental illness are *"highly stigmatized"*, *"viewed in negative ways" *and *"given derogatory labels". *Some participants noted that in the African setting, mental illness is strongly associated with disgrace and loss of respect in society. Many stakeholders highlighted how people perceive mentally ill patients as incapable and stupid, as reflected in this housing officer's remark:

"...*Unfortunately, people with mental illness are taken to be those who can not think for themselves; whatever they say they are mad, even if they improve... whatever they give...even if its good, we say they are mad...there is that ideology that if you are mental, then you don't have any idea"*.

Other respondents indicated that at the health facilities, if people come for mental health conditions, they are pinpointed at in a belittling manner. This was reported to also keep away some of the patients from seeking care:

"...*they call him 'mulalu' (he is mad). That is a negative attitude towards the patients. So, when one is called like that, he can not come back*"

(SSI, Mental health nurse)

Several respondents indicated that the psychiatric hospital itself is a major source of stigma, as one teacher explained:

'There are some places the stigma is there... remains... once you have seen the inside walls of the mental hospital, and you come back, I mean nothing about you is correct.....'

This widespread stigma appears to adversely affect people's willingness to seek appropriate care on one hand. On the other hand, families discourage their relatives from seeking care as they do not want to be associated with mental illness and its negative connotations.

## Discussion

This study provides qualitative insights into the help-seeking behaviours of individuals with mental health problems, and the factors that influence such behaviours, in the urban and rural communities in Uganda.

The results revealed that traditional healers are usually the first source of care people seek when faced with mental health problems, and frequently the only source of care sought. The findings are in line with results from a study by Basic Needs Uganda [16] and another study in Uganda [17] which both found that the majority of the patients with mental illness first visit traditional healers. Findings from studies in North Africa [18,19], East Africa [20], West Africa[21,22] and parallel findings in Sub-Saharan Africa [23-26] have all revealed the popularity and widespread use of traditional healers for psycho-social problems on the continent.

The results revealed that a multitude of cultural, social and economic factors shape the widespread use of traditional and faith healers. The cultural authority of traditional constructions of health and illness appears to play a significant part in the appeal of traditional mental healthcare systems. This corroborates findings from other studies which revealed that cultural perceptions of mental disorders as 'spiritual' illnesses may be a significant influencing factor in the popularity of traditional and faith healers [27-30]. It thus seems that the orientation of orthodox psychiatric services may be alienating and foreign for people with alternative beliefs and worldviews [31,32].

The type of care and treatment that is given surfaced as an additional factor influencing help-seeking behaviour, and the widespread use of traditional healers. The time and psychological and social support afforded by traditional practices play a role in their popularity, in contrast to the supposed hostile and rushed care provided at conventional mental health facilities. Although they are criticised for lack of scientific approach in their treatment modalities, this finding corroborates with results from other studies which highlighted the immense psychosocial support many traditional and faith healers provide; factors which are highly valued by their service users [27,30,33,34].

This study also revealed that that accessibility of health facilities and financial costs associated with care may also influence help-seeking behaviour. The inaccessibility of conventional mental health facilities, and thus the consequent high transport costs and other financial implications associated accessing such care were seen to deter individuals from such services. On the other hand, the cheap and readily available nature of traditional healers was seen to increase their appeal and usage. Indeed, Uganda has a high population growth rate, but a small number of mental health professionals, particularly in rural areas [12,32]. Such problems germane to orthodox psychiatric services are not unique to Uganda, but are insidious in many other low-income countries [35-37]. The results from this study thus substantiate findings from other studies in Africa that have suggested that the scarcity and high-cost of more conventional psychiatric mental health care may be in part responsible for their poor uptake [26,38,39]. For many poor people, also traditional healers may be the only affordable and accessible form of health care [40-42].

An interesting finding from this study was that people may not be aware of the availability of conventional mental health services, and may thus overestimate their inaccessibility and shortage. It thus seems that there is a blurring between actual available mental health services and perceptions around accessibility.

Finally, the widespread stigma surrounding mental illness and those affected was also seen to influence help-seeking behavior, by deterring many people from accessing care. This study revealed that the stigma surrounding mental illness is ubiquitous, findings which have been shown in another study in Uganda [16]. The results from this study highlighted how such stigma frequently demoralizes a person from accessing care, a phenomenon that has been widely acknowledged on a global scale [43-45].

It thus seems that a range of factors may be influencing the help-seeking behaviour of individuals in Uganda. Some of these factors relate to cultural and social norms residing within society. Other factors pertain to the nature of conventional mental health services and the numerous inadequacies of such facilities. This study has thus provided some insights into the gaps in conventional mental health service delivery, which can be used to guide the development of relevant services and policies

## Study Limitations

One major limitation of this study is the fact that family members of people with mental illness were not interviewed as a separate group of stakeholders, although several of the stakeholders interviewed were also family members of service users. Family members should be included in future in such research, as a key stakeholder group.

## Conclusion

The low uptake of mental health services in Uganda needs to be taken seriously, as this severely undermines recent efforts in the country aimed at improving the detection and treatment of mental disorders. The results suggest a need to strengthen the whole health system by providing adequate human and financial resources to conventional mental health services, and distributing such services so that they are more accessible to all populations, particularly in rural areas. Increasing the uptake of mental health services also requires addressing the widespread stigma surrounding mental illness and those affected. Finally, in order to start reaching more people, it is important to start taking the views and beliefs of the community more seriously, as well as placing more emphasis on increasing the dialogue with traditional healers. Indeed, beginning with the Alma Ata Declaration in 1978, and again in 2002, the World Health Organization has appealed to governments to start promoting the inclusion and integration of traditional practitioners in national and donor-specific health programmes [46,47]. This is essential in order to ensure that services and policies better meet the mental health needs of the country.

## Competing interests

The authors declare that they have no competing interests.

## Authors' contributions

JN is a Research Assistant on the project. He drafted the paper and he is the lead author.

SJ is the Research Officer on the project. He has been involved in editing of this paper.

FK is the Principal Investigator of the Ugandan site of the MHaPP. He participated in the design of the study, and data analysis He has been actively involved in the editing of this paper. DK is a Research Assistant. She conceived the idea and also took part in editing and alignment of the paper. AJF is the Project Director; He participated in editing and alignment of this paper. SC is a Research Officer in the Mental Health and Poverty Project. She gave overall advice and guidance for the paper, and actively participated in editing and alignment. The listed MHaPP group members conceived this study and were jointly involved in designing the data collection and analysis methods.

## Authors' information

JN is a clinical psychologist at Butabika National Referral Mental Hospital. He is also a Research Assistant on Mental Health and Poverty Project.

JS is a Clinical Psychologist and is currently a Research Officer on the Mental Health and Poverty Project. He is also a part-time Assistant Lecturer in the Department of Mental Health and Community Psychology, Makerere University.

FK is a Senior Consultant Psychiatrist and Executive Director of Butabika National Referral Mental Hospital, Uganda. He is the Ugandan Principal Investigator on the Mental Health and Poverty Project. He is also the President Uganda Psychiatric Association, and formerly WPA

zone 14 (East and Southern Africa) Representative.

DK is a Senior Clinical Psychologist at Butabika National Referral Mental Hospital, Uganda. She is currently pursuing Doctoral studies at Norwegian University of Science and Technology.

SN is a Public Health Specialist and the Principal Medical Officer in charge of mental health at the Ministry of Health headquarters in Uganda.

AJF is a professor of child and adolescent psychiatry, Department of Psychiatry and Mental Health, University of Cape Town, South Africa. He is the director of MHAPP.

SC is a Research Officer in the Mental Health and Poverty Project, Department of Psychiatry and Mental Health, University of Cape Town, South Africa. She holds a Masters degree in Public Health from the same University.

## References

[B1] Okello ES Explanatory Models and Help-Seeking Behavior: Pathways to Psychiatric Care Among Patients Admitted for Depression in Mulago Hospital, Kampala, Uganda Qualitative Health Research 2007 17 1 14 25 10.1177/1049732306296433 17170240

[B2] Howerton A Byng R Campbell J Hess D Owens C Aitken P Understanding help seeking behaviour among male offenders: qualitative interview study British Medical Journal 2007 334 7588 303 10.1136/bmj.39059.594444.AE 17223630 PMC1796718

[B3] Bebbington PE Meltzer H Brugha TS Unequal access and unmet need: neurotic disorders and use of primary care services Psychological Medicine 2000 30 1359 1367 10.1017/S0033291799002950 11097076

[B4] Abbo C Management of Mental Health Problems by Traditional Healers in Kampala District 2003 Kampala: Department of Psychiatry. Kampala, Makerere University

[B5] Abbo C Profiles and outcome of traditional healing practices for severe mental illnesses in two districts in Eastern Uganda 2009 Stockholm and Kampala: Karolinska Institutet and Makerere University 10.3402/gha.v4i0.7117 PMC3150106 21845144

[B6] Angermeyer MC Matschinger H Riedel-Heller SG What to do about mental health disorder-help seeking recommendations of the lay public Acta Psychiatrica Scandinavica 2001 103 220 225 10.1034/j.1600-0447.2001.103003220.x 11240579

[B7] Ministry of Health Health Sector Strategic Plan I 2000 Kampala, Uganda: Ministry of Health

[B8] Ministry of Health Health Sector Strategic Plan II 2005 Kampala, Uganda: Ministry of Health

[B9] Ndyanabangi S Basangwa D Lutakome J Mubiru C Uganda mental health country profile International Review of Psychiatry 2004 16 1-2 54 62 10.1080/09540260310001635104 15276938

[B10] Flisher AJ Lund C The mental health and poverty project: Some preliminary findings Mental Health Reforms 2009 1 11 13

[B11] WHO, & Wonca Integrating Mental Health into Primary care: A Global Perspective 2008 Singapore: World Health Organisation press

[B12] Ssanyu R Mental Illness and Exclusion: Putting Mental Health on the Development Agenda in Uganda 2007 Policy brief 2, Kampala, Uganda: Chronic Poverty Research Centre http://www.chronicpoverty.org/uploads/publication_files/CPRC-UG_PB_2007-2.pdf

[B13] UBOS Uganda Population Report 2006 2006 Kampala: Uganda Bureau of Statistics

[B14] Kigozi F Ssebunnya J Kizza D Ndyanabangi S Green A Maye O Bird P Funk M Faydi E Drew N Lund C Flisher A A situational analysis of the Mental health Systems: A PHASE 1 COUNTRY REPORT 2008 http://workhorse.pry.uct.ac.za:8080/MHAPP/public/public/resources/Uganda_report Retrieved June 21, 2010, from MentalHhealth and Poverty Project

[B15] Ritchie J Spencer L Bryman, Burgess Qualitative data analysis for applied policy research Analysing qualitative data 1994 London: Routledge 173 194 full_text

[B16] Basic Needs Uganda Social Stigma and Mental Health in Uganda 2005 Basic Needs U.K, Uganda

[B17] Teuton J Dowrick C Bentall RP How healers manage the pluralistic healing context: The perspective of indigenous, religious and allopathic healers in relation to psychosis in Uganda Social Science and Medicine 2007 65 6 1260 1273 10.1016/j.socscimed.2007.03.055 17521791

[B18] Alem A Araya JM Kebede D Kullgren G How are mental disorders seen and where is help sought in a rural Ethiopian community? A key informant study in Butajira, Ethiopia Acta Psychiatr Scand 1999 100 40 47 10.1111/j.1600-0447.1999.tb10693.x 10470354

[B19] Bekele YY Flisher AJ Alem A Bahiretebeb Y Pathways to psychiatric care in Ethiopia Psychological Medicine 2009 39 475 483 10.1017/S0033291708003929 18606050

[B20] Kilonzo GP Simmons N Development of mental health services in Tanzania: A reappraisal for the future Social Science and Medicine 1998 47 4 419 428 10.1016/S0277-9536(97)10127-7 9680226

[B21] Franklin RR Sarr D Gueye M Sylla O Collignon R Cultural response to mental illness in Senegal: Reflection through patient companions - part 1. Methods and descriptive data Social Science and Medicine 1996 42 3 325 338 10.1016/0277-9536(95)00108-5 8658228

[B22] Gureje O Lasebikan V Use of mental health services in a developing country Social Science and Medicine 2006 41 1 44 10.1007/s00127-005-0001-7 16341828

[B23] Crawford AT Lipsedge M Seeking help for psychological distress: The interface of Zulu traditional healing and Western biomedicine Mental Health, Religion & Culture 2004 7 2 131 148

[B24] Mayeya J Chazulwa R Mayeya P Mbewe E Mwape-Magolo L Kasisi F Bowa AC Zambia mental health country profile International Review of Psychiatry 2004 16 1-2 63 72 10.1080/09540260310001635113 15276939

[B25] Patel V Simunyu E Gwanzura F The pathways to primary mental health care in high-density suburbs in Harare, Zimbabwe Social Psychiatry and Psychiatric Epidemiology 1997 32 97 103 10.1007/BF00788927 9050351

[B26] Campbell-Hall V Petersen I Bhana A Mjadu S Hosegood V Flisher AJ Collaboration between traditional practitioners and primary health care staff in South Africa: developing a workable partnership for community mental health services Transcultural Psychiatry 2010 47 4 610 628 10.1177/1363461510383459 20940271

[B27] Hewson MG Traditional healers in southern Africa Annals of Internal Medicine 1998 128 12 1029 1034 9625666 10.7326/0003-4819-128-12_part_1-199806150-00014

[B28] Jahoda G Ademuwagun Z, Ayoade JA, Harrison IE, Warren DM Traditional healers and other institutions concerned with mental illness in Ghana African therapeutic systems 1979 Waltham: African Studies Association 98 109

[B29] Ofori-Atta AML Linden W The effect of social change on causal beliefs of mental disorders and treatment preferences in Ghana Social Science and Medicine 1995 40 9 1231 1242 10.1016/0277-9536(94)00248-R 7610429

[B30] Tanner RES Concern, cooperation, and coexistence in healing British Medical Journal 1999 319 133 10398661 10.1136/bmj.319.7202.133 PMC1116217

[B31] Leff J Social Inclusion of people with Mental illness 2006 New York: Cambridge University Press

[B32] Ministry of Health A baseline Survey of the Mental Health Situation in 11 Districts in Uganda 2004 Support to Health Sector Strategic Plan Project. Kampala, Uganda: Ministry of Health

[B33] Meissner O The traditional healer as part of the primary health care team? South African Medical Journal 2004 94 901 902 15587451

[B34] Van der Geest S Is there a role for traditional medicine in basic health services in Africa? A plea for community perspective Tropical Medicine and International Health 1997 2 9 903 911 10.1046/j.1365-3156.1997.d01-410.x 9315049

[B35] Jacob KS Sharan P Mirza I Garrido-Cumbrera M Seedat S Mari JJ Sreenivas V Saxena S Mental health systems in countries: Where are we now? The Lancet 2007 370 1061 1077 10.1016/S0140-6736(07)61241-0 17804052

[B36] Kohn R Saxena S Levav I Saraceno B The treatment gap in mental health care Bulletin of the World Health Organization 2004 82 858 866 15640922 PMC2623050

[B37] Saxena S Thornicroft G Knapp M Whiteford HA Resources for mental health: Scarcity, inequity and inefficiency Lancet 2007 370 878 889 10.1016/S0140-6736(07)61239-2 17804062

[B38] Roberts H A way forward for mental health care in Ghana? Lancet 2001 357 1859 10.1016/S0140-6736(00)05020-0 11410206

[B39] Tsey K Traditional medicine in contemporary Ghana: A public policy analysis Social Science and Medicine 1997 45 7 1065 1074 10.1016/S0277-9536(97)00034-8 9257398

[B40] Cocks M Moller V Use of indigenous and indigenized medicines to enhance personal well-being: A South African case study Social Science and Medicine 2002 54 3 387 398 10.1016/S0277-9536(01)00037-5 11824915

[B41] Sodi T Towards recognition of indigenous healing: Prospects and constraints Journal of Comprehensive Health 1996 7 1 5 9

[B42] Tabi M Nursing in Ghana Journal of Nursing Administration 1994 24 9 17 18 8089712

[B43] Corrigan PW Watson AC Understanding the impact of stigma on people with mental illness World Psychiatry 2002 1 16 20 16946807 PMC1489832

[B44] Baumann AE Stigmatization, social distance and exclusion because of mental illness: The individual with mental illness as a 'stranger' International Review of Psychiatry 2007 19 2 131 135 10.1080/09540260701278739 17464791

[B45] Botha UA Koen L Niehaus DJH Perceptions of a South African schizophrenia population with regards to community attitudes towards their illness Social Psychiatry and Psychiatric Epidemiology 2006 41 619 623 10.1007/s00127-006-0071-1 16733630

[B46] Summerton JV The organisation and infrastructure of the African traditional healing system: Reflections from a sub-district of South Africa African Studies 2006 65 2 297 319 10.1080/00020180601035708

[B47] Wreford J '*Sincedisa *- we can help!' A literature review of current practice involving traditional African healers in biomedical HIV/AIDS interventions in South Africa Social Dynamics 2005 31 2 90 117 10.1080/02533950508628709

